# A Clinical Decision Support System for Post-Surgical Cardiovascular Remote Monitoring

**DOI:** 10.3390/clinpract16050093

**Published:** 2026-05-15

**Authors:** Charalampia Pylarinou, Francesk Mulita, Efstratios Koletsis, Vasileios Leivaditis, Elias Liolis, Lefteris Gortzis, Dimosthenis Mavrilas

**Affiliations:** 1 Department of Mechanical and Aeronautical Engineering, University of Patras, 26504 Patras, Greece; mauril@mech.upatras.gr; 2 Department of Surgery, University Hospital of Patras, 26504 Patras, Greece; 3 Department of Cardiothoracic Surgery, University Hospital of Patras, 26504 Patras, Greece; ekoletsis@upatras.gr; 4 Department of Cardiothoracic Surgery, Westpfalz-Klinikum, 67655 Kaiserslautern, Germany; vnleivaditis@gmail.com; 5 Department of Oncology, University Hospital of Patras, 26504 Patras, Greece; lioliselias@yahoo.gr; 6 Research & Development, CAREPOI™, 26221 Patras, Greece; gortzis@med.upatras.gr

**Keywords:** retrieval-augmented generation, clinical decision support, evidence-based medicine, artificial intelligence, cardiovascular surgery, remote monitoring

## Abstract

**Background:** Post-surgical cardiovascular monitoring places a heavy information burden on clinical teams, requiring the rapid synthesis of patient history, intraoperative data, monitoring streams, and surgical outcome evidence. Existing clinical decision support systems handle this integration poorly, and most offer little visibility into their reasoning. We present a Retrieval-Augmented Generation (RAG) architecture designed specifically for this domain, with a focus on evidence traceability and practical workflow integration. **Methods:** We describe a three-layer RAG architecture comprising a retrieval layer that creates 768-dimensional representations of clinical scenarios; an augmentation layer using a stacking ensemble (Random Forest and XGBoost base learners with a logistic-regression meta-learner) to integrate patient-specific data with retrieved evidence and produce calibrated probability estimates; and a generative layer using a fine-tuned BERT classifier together with Gemini 2.5 Pro to synthesise actionable clinical recommendations. Components were prototyped on publicly available, de-identified data from MIMIC-III and the MIMIC-III-Ext-PPG benchmark to verify pipeline integrity. **Proposed Evaluation Framework:** This paper presents a system architecture rather than a clinically validated implementation. We outline a structured evaluation framework to assess the technical performance and clinical applicability of the RAG architecture, encompassing the technical validation of system components, expert assessment of clinical workflow integration potential, and analysis of interpretability features essential for healthcare deployment. Specific technical targets include retrieval precision >90% for relevant evidence, query response time <3 s, and a clinical appropriateness rating of >85% from expert review. **Conclusions:** We describe a RAG architecture for post-surgical cardiovascular monitoring in which every recommendation is linked to retrievable source documents, making the reasoning visible and challengeable. A structured evaluation framework is proposed to guide the system towards clinical validation.

## 1. Introduction

Post-surgical cardiovascular care generates an information burden that current clinical decision support systems (CDSS) handle poorly [1,2]. At the bedside, clinicians must rapidly reconcile patient history, intraoperative events, real-time monitoring streams, and the growing surgical outcome literature—tasks that rule-based or opaque machine learning tools support only in part [3]. When these tools do offer guidance, their reasoning is rarely visible, and this lack of transparency remains a practical barrier to adoption in cardiac surgery units [4].

Retrieval-Augmented Generation (RAG) offers a different approach. Rather than encoding all knowledge in model weights, RAG queries a curated evidence base at inference time, so that each recommendation is grounded in retrievable, inspectable source documents [5]. This traceability is important for clinical adoption: a surgeon needs not just a risk estimate but a visible reasoning chain that can be examined and, if necessary, challenged [6].

Deploying RAG in a clinical setting introduces its own constraints. Workflow integration, evidence quality control, and regulatory audit requirements all shape what an acceptable system looks like in practice [7]. In cardiac surgery, the pace of postoperative deterioration means that response latency matters as much as classification accuracy [8]. The postoperative monitoring component of our system drew on the DETER algorithm [9,10,11,12,13,14,15,16], which showed that combining device-based telemetry with generative AI can produce actionable deterioration warnings early enough to alter management. Recent studies confirm that continuous wireless telemetry after cardiac surgery detects arrhythmias earlier than intermittent monitoring [17], and wearable-based early warning scores show promising performance as part of post-surgical escalation systems [18].

Existing cardiovascular remote monitoring tools can be broadly grouped along a few lines, each with characteristic strengths and limitations. Static risk calculators such as EuroSCORE II and the STS Predicted Risk of Mortality and Morbidity provide a preoperative estimate that is not designed to update as postoperative data accrue. Continuous telemetry platforms detect arrhythmias and haemodynamic instability earlier than intermittent monitoring, but each signal is typically interpreted in isolation rather than against published surgical outcome evidence or prior similar cases. Machine learning early-warning systems built on physiological streams produce risk scores that often perform well, yet they rarely expose the clinical evidence on which a given alert rests, which can leave clinicians to accept or override a relatively opaque output. The architecture proposed here is intended to complement rather than replace these tools: it updates risk continuously from monitoring data while retrieving and citing the surgical outcome literature, guideline statements, and institutional cases that justify each recommendation. We are not aware of an existing cardiovascular remote monitoring system that combines retrieval-grounded reasoning with continuous postoperative data integration in this way [19,20,21,22,23].

We describe a purpose-built RAG architecture for post-surgical cardiovascular monitoring. The central design goal was traceability: every recommendation the system produces can be linked back to the specific retrieved evidence that generated it. A secondary goal was realistic integration with the perioperative workflows already in place at participating centres.

The study pursued five objectives: (1) design a RAG architecture suited to cardiovascular surgery decision support; (2) build domain-specific retrieval and evidence integration components; (3) make the reasoning behind AI-generated recommendations fully auditable; (4) propose an evaluation framework appropriate for a system at this stage of development; and (5) assess its potential to augment clinical decision-making while preserving physician oversight.

## 2. Methods

### 2.1. RAG Architecture Overview

**Figure 1:**
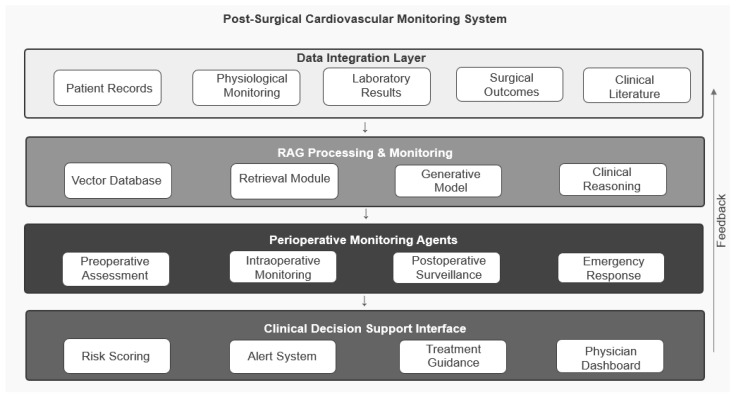
System architecture of the RAG-based clinical decision support system, depicting four functional bands—Data Integration, RAG Processing and Monitoring, Perioperative Monitoring Agents, and Clinical Decision Support Interface—with feedback flow between bands. The three-layer RAG core sits inside the RAG Processing and Monitoring band: the Retrieval Module performs retrieval; Clinical Reasoning performs augmentation (ensemble integration, conflict surfacing, patient-specific contextualisation); and the Generative Model performs output synthesis, all supported by the Vector Database.

The proposed RAG architecture comprises three interconnected RAG layers—retrieval, augmentation, and generative—operating within a broader four-band system architecture (Data Integration, RAG Processing and Monitoring, Perioperative Monitoring Agents, Clinical Decision Support Interface) shown in Figure 1. The architecture integrates domain-specific knowledge sources, employs clinical workflow-aware processing, and is designed to support interpretability throughout the decision support process.

**Stage of work.** This paper describes a system architecture and a proposed evaluation framework. Components of the pipeline have been prototyped using publicly available, de-identified clinical data—specifically the MIMIC-III Cardiac Surgery Recovery Unit (CSRU) cohort [21] for clinical text and structured outcomes, complemented by the MIMIC-III-Ext-PPG waveform benchmark [22] for telemetry-style signal processing—to verify pipeline integrity. The system has not been trained on prospectively collected, multi-centre cardiovascular surgery outcome data from participating clinical sites, has not been validated against prospective clinical endpoints, and is not deployed at any clinical site. Sentences in the following sections that describe model behaviour or component function should be read as descriptions of the designed pipeline rather than as reports of empirical performance, except where prototype-stage evaluation is explicitly noted.

The system operates through a systematic workflow where clinical queries trigger retrieval of relevant evidence from multiple knowledge sources, followed by context-aware integration and augmentation of this evidence with patient-specific data, culminating in the generation of interpretable clinical recommendations with full evidence provenance.

**Figure 2:**
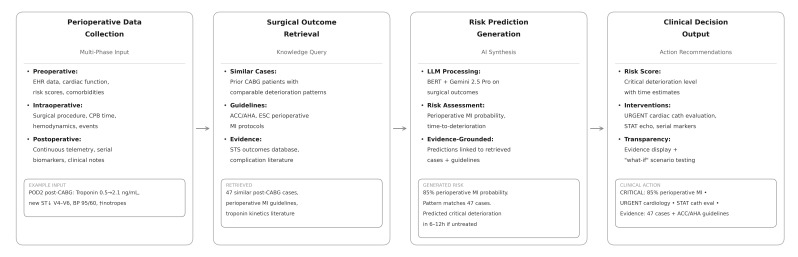
End-to-end RAG pipeline applied to a worked post-CABG case, showing the four operational stages: Perioperative Data Collection, Surgical Outcome Retrieval, Risk Prediction Generation, and Clinical Decision Output. Numbers shown are illustrative for a hypothetical case and not derived from a deployed system.

The end-to-end pipeline is illustrated in Figure 2, applied to a worked post-CABG case. Each layer has a defined input and output, and the interfaces between them are designed so that individual components can be tested and updated independently.

### 2.2. Input Layer

**Figure 3:**
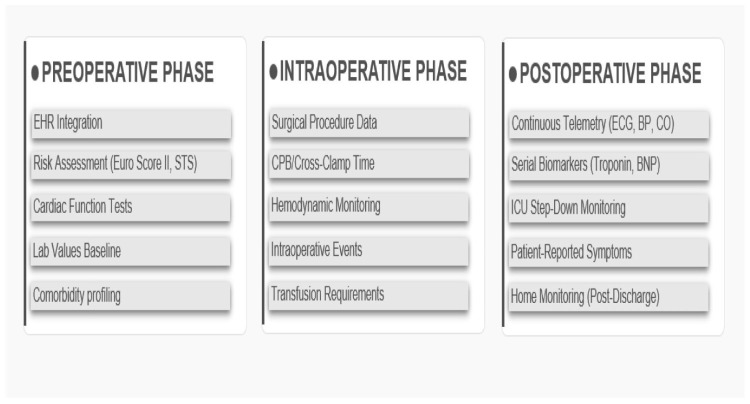
Multi-phase data sources for the post-surgical cardiovascular monitoring RAG system, organised by surgical phase (preoperative, intraoperative, postoperative). Detailed in Table 1.

This layer is responsible for collecting comprehensive data streams across the surgical continuum, organised by surgical phase as shown in Figure 3 and detailed in Table 1.

Such data are drawn from the CAREPOI (™) perioperative telemetry monitoring system [9]; the system connects to hospital HIS/EHR and intensive care unit (ICU) monitoring systems via HL7/Fast Healthcare Interoperability Resources (FHIR) interoperability standards, where applicable. Patient-reported symptoms during recovery are processed via two NLP components with distinct, partly overlapping roles. cTAKES (Apache Clinical Text Analysis and Knowledge Extraction System) [20] provides the primary structured extraction: named entity recognition with direct mapping to standardised terminologies (UMLS, SNOMED CT, RxNorm) for symptoms, medications, procedures, and anatomical sites, together with negation detection and temporal reasoning, applied to free text in EHRs [10]. A BioBERT [19]-based NER model—a BERT variant pre-trained on biomedical literature, fine-tuned for symptom-mention recognition—runs in parallel, primarily on patient-narrative text and telehealth consultation transcripts, where vocabulary, grammar, and abbreviations diverge from clinical-note conventions and are therefore poorly recovered by dictionary-based extraction [23]. cTAKES output forms the primary structured representation passed to downstream layers; BioBERT extractions that do not align with a cTAKES concept are flagged for clinician review and as candidates for dictionary expansion. The two components together combine the precision and standardised-terminology mapping of cTAKES with the contextual recall of a learned model on free-form patient input.

A rule-based system incorporating established post-surgical complication signatures—implemented using HL7 FHIR Clinical Reasoning (specifically, the FHIR ClinicalImpression, ClinicalReasoning, and PlanDefinition resources)—flags abnormal parameter combinations for clinician review. This approach encodes published clinical guidelines (e.g., STS/ACC/AHA post-cardiac surgery complication criteria, ESC atrial fibrillation guidelines) as computable clinical decision logic within FHIR-native rule sets, enabling deterministic, interpretable, and standard-compliant identification of high-risk parameter constellations such as new-onset arrhythmia combined with troponin elevation, or fever with wound erythema and leukocytosis. The rule engine and the time-series encoder described below operate as parallel preprocessing branches alongside the clinical-text components above; their outputs are combined in the augmentation layer rather than chained.

On the physiological-signal side, a Transformer-based time-series encoder is used to produce learned representations of the continuous monitoring stream—vital signs, waveforms, and serial laboratory values—across the perioperative timeline. Transformer architectures are appropriate to this setting because they capture long-range temporal dependencies [24] and parallelise efficiently on the high-frequency data typical of ICU monitoring. Together with the structured concept extractions from cTAKES/BioBERT-NER and the alerts from the FHIR rule engine, this gives the augmentation layer three temporally aligned but methodologically distinct views of the patient—deterministic, learned-textual, and learned-physiological—which together form the input representation used for deterioration modelling.

BioBERT-based medical entity identification is used for entity recognition and sentence segmentation, abbreviation expansion, and negation detection as part of clinical text preprocessing [23]. Transformers are highly parallelisable, making them computationally efficient for processing high-frequency continuous monitoring streams, especially in real-time intensive care unit (ICU) environments. Together, these preprocessing steps give the DSS a structured, temporally ordered data representation—a prerequisite for accurate deterioration modelling, and a holistic, temporal understanding of the patient’s surgical journey, crucial for accurate deterioration prediction [10].

### 2.3. Retrieval Layer Design

The knowledge base used by the retrieval layer draws on three main sources: peer-reviewed cardiovascular surgery literature, anonymised institutional outcome data from participating centres, and current clinical practice guidelines. Combining these sources improves the specificity and clinical relevance of retrieved evidence across the perioperative continuum, including procedure-specific profiles for 97 cardiovascular surgical procedures [11].

Using provided application programming interfaces (APIs), the retrieval layer queries relevant internal surgical outcome databases and external cardiovascular literature in response to patient-specific deterioration risk queries. Pre-processing pipelines normalise diverse data formats (JSON, HL7, and FHIR) allowing seamless interoperability across perioperative systems. Dense vector search techniques using Sentence-BERT embeddings enable fast, semantically accurate query resolution [12].

The system employs domain-adapted BERT models to create 768-dimensional vector representations of clinical scenarios and knowledge base content [13]. The embedding process involves Clinical Query Processing, Knowledge Base Vectorisation, and a Retrieval Algorithm.

When the system generates a deterioration prediction, it simultaneously presents the “*top-k relevant records*” (e.g., similar surgical cases, complication management guidelines, outcome literature) that informed its assessment. The RAG architecture allows clinicians to see the factual basis for the deterioration prediction, enabling them to scrutinise the internal logic and verify its alignment with their own clinical reasoning and established cardiovascular surgery evidence. This directly addresses the “*black box*” problem of many AI systems in critical care. This design choice is intended to ensure that the DSS’s outputs are not just statistical predictions but are grounded in verifiable surgical outcome evidence, fostering trust and explainability [14]. Implementation involves building and maintaining robust search indexes of surgical outcomes and embedding models trained on cardiovascular surgery literature.

The retrieval component implements semantic search across cardiovascular surgery literature, institutional outcome databases, and clinical practice guidelines to identify contextually relevant information for patient-specific risk assessment. The system employs dense vector representations using Sentence-BERT embeddings to encode patient presentations and retrieve similar prior cases from the indexed surgical knowledge bases. ClinicalBERT, trained on MIMIC-III clinical notes, may additionally be deployed for clinical-note-specific retrieval tasks. Knowledge base construction encompasses multiple complementary data sources including peer-reviewed cardiovascular surgery literature from PubMed.

Vector embedding generation uses domain-adapted BERT models pre-trained on cardiovascular surgery literature to create 768-dimensional representations of clinical scenarios. Patient presentations undergo systematic preprocessing including medical entity extraction, temporal sequence identification, and clinical context normalisation before embedding generation [24].

The retrieval process uses cosine similarity scoring to identify relevant surgical outcomes, complication patterns, and evidence-based intervention strategies. Similarity threshold optimisation aims to balance high-quality evidence retrieval with computational efficiency. The system incorporates temporal relevance weighting to prioritise recent evidence while maintaining access to foundational clinical knowledge. A mechanism is planned to validate retrieved evidence through automated fact-checking against established clinical databases and expert-curated knowledge bases.

### 2.4. Augmentation Layer Implementation

As an illustrative case, consider a 68-year-old patient with prior myocardial infarction and previous coronary artery bypass grafting (CABG) on dual antiplatelet therapy who presents for non-cardiac surgery. The architecture surfaces this profile’s elevated complication risk and triggers higher-intensity perioperative monitoring rather than relying on a single global risk score. The retrieved evidence undergoes systematic integration and contextualisation through a stacking ensemble trained on cardiovascular surgery outcome data. The ensemble uses Random Forest and XGBoost as base learners with a logistic-regression meta-learner combining their predictions, producing calibrated probability estimates with confidence intervals for procedure-specific complications [15]. The augmentation step combines patient-specific risk factors, surgical complexity, institutional benchmarks, and guideline recommendations to produce individualised risk profiles and ranked intervention options. For example, a patient with prior myocardial infarction undergoing CABG would receive a risk profile reflecting their elevated baseline cardiovascular risk, the surgical complexity of revascularisation, the participating centre’s CABG outcome data, and current STS/AHA guideline recommendations. Training of the stacking ensemble is planned on expert-annotated cardiovascular surgery outcomes once such data become available.

Context-aware filtering removes irrelevant or contradictory information while preserving clinically significant evidence. Multi-source evidence reconciliation identifies consistent patterns across different knowledge sources and highlights potential conflicts requiring clinical judgement.

Patient-specific contextualisation adjusts general clinical evidence to account for individual patient characteristics including age, comorbidities, surgical complexity, and institutional factors. Personalisation algorithms weight evidence relevance based on similarity to the current patient’s clinical profile (e.g., for a patient on dual antiplatelet therapy, prior cases on dual rather than single therapy receive greater weight in bleeding-risk estimation). The augmentation layer generates structured clinical insights including risk factor identification, complication probability estimates, evidence-based intervention recommendations, and monitoring intensity suggestions.

Three features distinguish this augmentation layer from a standard RAG implementation. First, the retrieved evidence is not passed directly to the generator; it is first integrated through the stacking ensemble described above, producing calibrated probability estimates with confidence intervals for procedure-specific complications (myocardial infarction, atrial fibrillation, low cardiac output syndrome, sternal wound infection) rather than free-text prose alone. Second, contextualisation is temporal as well as patient-specific: retrieved evidence is reweighted according to the postoperative day, because the differential for chest pain, fever, or new arrhythmia shifts substantially between the immediate postoperative period, the step-down phase, and the post-discharge recovery window. Third, multi-source evidence reconciliation surfaces conflicts between sources—for example, between an institutional outcome record and a more recent society guideline—rather than silently selecting one, so that clinical judgement, not the retriever’s similarity score, resolves the disagreement.

### 2.5. Generative Layer Architecture

**Model Roles.** Three distinct model families serve distinct functions in the pipeline, and we distinguish them explicitly to avoid conflating retrieval with generation. (i) Sentence-BERT, with ClinicalBERT optionally applied for clinical-note retrieval, encodes queries and documents them as 768-dimensional dense vectors for similarity search; these are encoder-only models and are used purely for retrieval, not for generation. (ii) A domain-fine-tuned BERT classifier in the augmentation layer produces calibrated probability estimates for procedure-specific complications from the retrieved evidence; this is also discriminative, not generative. (iii) Gemini 2.5 Pro is the only generative component: it receives the top-k retrieved evidence together with the augmentation layer’s structured outputs as a prompt, and synthesises a natural-language clinical recommendation that cites the retrieved sources. The generative output is grounded in the patient’s specific surgical context—preoperative risk factors, intraoperative course, and current postoperative trajectory—and aligned with validated post-surgical complication patterns to maintain clinical relevance.

The synthesised recommendations include risk stratification scores for specific complications (myocardial infarction, arrhythmias, heart failure, infection, bleeding), clinical reasoning outputs explaining the prediction basis, and diagnostic/intervention suggestions.

**Hallucination control.** Hallucination control rests on three mechanisms. The generator’s prompt is restricted to retrieved, citable content, and generation is suppressed when retrieval similarity falls below the threshold. Each factual claim in the generated output is post hoc-verified against the cited sources. When generation is suppressed or fails verification, the system falls back to the augmentation layer’s structured output—a probability estimate with ranked intervention list—rather than returning nothing.

**Training.** The current prototype is trained on MIMIC-III CSRU records [21] using mortality and ICD (International Classification of Diseases)-coded major-adverse-event labels, with waveform features incorporated from the MIMIC-III-Ext-PPG benchmark [22]; this prototype training serves to verify pipeline integrity rather than produce a clinically deployable model. Multi-centre training with the full label set—30-day mortality, major adverse cardiac events, readmission, and functional recovery—is planned once data-sharing agreements with participating clinical sites are in place. Supervised fine-tuning combined with reinforcement learning from human feedback is the planned alignment approach.

The generative component is designed to produce structured clinical recommendations including risk stratification scores with confidence intervals, evidence-based intervention recommendations with supporting citations, and monitoring intensity guidelines tailored to predicted risk levels—for example, a high postoperative atrial fibrillation risk profile triggering 48 h continuous telemetry with prophylactic amiodarone consideration per the relevant ESC guideline, alongside anticipated complication signatures with temporal progression patterns.

Key configuration parameters across the three layers are summarised in Table 2.

### 2.6. Decision Support Integration

The system provides tailored clinical suggestions including urgent diagnostic testing recommendations (repeat ECG, echocardiography, cardiac catheterisation), medication adjustments (inotropes, antiarrhythmics, diuretics), intensification of monitoring frequency, specialist consultation recommendations (cardiology, cardiac surgery), and transfer to higher level of care (step-down to ICU).

**Alert fatigue mitigation.** The decision support layer is designed to mitigate alert fatigue through three mechanisms. First, recommendations are tiered by severity: only predictions exceeding clinically actionable thresholds (e.g., calibrated complication probability above a unit-defined cut-off, or evidence of acute deterioration) trigger active alerts, while lower-acuity findings are surfaced only when the clinician reviews the patient dashboard. Second, predictions with retrieval similarity below the configured threshold or with failed post hoc claim verification (described in the hallucination control mechanism above) are suppressed rather than presented as low-confidence alerts, so that the clinician’s attention is reserved for outputs that are both grounded and confident. Third, per-unit alert ceilings and clinician-configurable preferences (e.g., suppression of recurring alerts on the same finding within a defined window) prevent alert saturation in busy postoperative settings, with a planned audit log to allow a retrospective review of suppressed events.

## 3. Evaluation Methodology

The objective of this evaluation methodology is to provide a basic framework for assessing the proposed RAG-based clinical decision support system, recognising that comprehensive clinical validation would require extensive real-world deployment and outcome measurement beyond the scope of this initial system design study. Given the absence of actual clinical results at this stage, the evaluation framework focuses on technical feasibility assessment, expert validation of system components, and preliminary analysis of clinical applicability.

### 3.1. Technical Component Validation

The evaluation begins with a systematic assessment of each RAG architecture component to ensure technical robustness and clinical relevance. For the retrieval layer, evaluation focuses on knowledge base completeness and quality, assessing the coverage of major cardiovascular surgery guidelines and literature, currency of evidence sources, and accuracy of semantic similarity matching. Expert cardiovascular surgeons would review sample retrievals to validate the clinical relevance and appropriateness of returned evidence for specific clinical scenarios. Response time benchmarking is intended to confirm compatibility with clinical workflow requirements, targeting sub-3 s response times for routine queries.

The augmentation layer undergoes evaluation for evidence integration quality and patient-specific contextualisation accuracy. Clinical experts assess the appropriateness of evidence filtering, contradiction resolution mechanisms, and personalisation algorithms through a review of sample cases representing diverse patient profiles and clinical scenarios. Inter-rater reliability assessment among clinical reviewers provides quantitative validation of system performance consistency. The generative layer evaluation emphasises the clinical appropriateness and safety of generated recommendations, with expert panels reviewing sample outputs for medical accuracy, completeness, and alignment with established clinical guidelines.

### 3.2. Interpretability and Trust Assessment

A critical evaluation component focuses on the system’s interpretability features, essential for clinical adoption and regulatory compliance. Clinical users evaluate evidence provenance completeness, reasoning chain clarity, and confidence scoring accuracy through structured review sessions. The assessment examines whether clinicians can effectively trace recommendation origins, understand the logical basis for AI-generated suggestions, and appropriately weight recommendations based on confidence indicators. Expert evaluation of alternative option presentation aims to confirm that the system surfaces multiple options rather than singular recommendations.

Interpretability is operationalised through structured rating sessions using a 5-point Likert scale across three dimensions—evidence provenance completeness, reasoning chain clarity, and citation completeness—applied by approximately three independent expert raters per case. Inter-rater agreement is reported as Cohen’s κ; a κ of ≥0.6 (substantial agreement, per Landis & Koch, 1977 [25]) is treated as the minimum threshold for a reliable rating. A composite mean rating of ≥4 of 5 across the three dimensions, in ≥80% of evaluated cases, is the predefined criterion for the system meeting its interpretability target.

A comparative analysis against existing clinical decision support tools provides context for system performance evaluation. While full clinical outcome comparison requires extensive prospective studies, initial assessment can compare recommendation quality, evidence citation completeness, and user interface effectiveness against established cardiovascular surgery risk assessment tools such as EuroSCORE II and STS risk calculators.

### 3.3. Clinical Workflow Integration Analysis

Preliminary workflow integration assessment examines system compatibility with existing clinical processes and electronic health record systems. Healthcare informatics specialists and clinical users review system interfaces, data integration requirements, and workflow modification needs through structured evaluation sessions. This assessment identifies potential implementation barriers, training requirements, and technical infrastructure needs for successful deployment.

User experience evaluation with cardiovascular surgeons, cardiologists, and critical care physicians provides insights into system usability, clinical relevance, and acceptance potential. Through controlled demonstration sessions using representative clinical scenarios, evaluators assess system response appropriateness, interface intuitiveness, and perceived clinical value. Qualitative feedback gathering identifies refinement priorities and implementation considerations for future deployment phases.

### 3.4. Safety and Risk Assessment

Given the high-stakes nature of post-surgical cardiovascular care, safety assessment constitutes a fundamental evaluation component. Expert clinical panels review system outputs for potential adverse recommendations, contraindicated suggestions, or clinically inappropriate guidance. Safety evaluation protocols include the assessment of recommendation conservatism, appropriate uncertainty expression, and robust handling of edge cases or unusual clinical presentations.

Risk assessment examines system robustness under various conditions including incomplete data, conflicting evidence sources, and atypical patient presentations. Technical risk evaluation addresses system reliability, data security, and privacy protection mechanisms essential for healthcare deployment. This preliminary safety assessment provides a foundation for more comprehensive risk management protocols required for clinical implementation.

### 3.5. Expected Evaluation Outcomes

The expected outcomes are as follows: validation that each architectural component functions within specification; expert confirmation that retrieved evidence is clinically relevant; and identification of the infrastructure and training requirements for site deployment. Specific technical targets are retrieval precision >90% for relevant evidence, query response time <3 s, and a clinical appropriateness rating of >85% from an expert review of the generated recommendations.

Clinical assessment outcomes focus on interpretability validation, workflow compatibility confirmation, and the identification of user training and support requirements. Safety evaluation establishes preliminary risk profiles and identifies additional safeguards needed for clinical deployment. The evaluation provides a foundation for future clinical validation studies while demonstrating system readiness for pilot implementation in controlled clinical environments.

## 4. Discussion

### 4.1. Clinical Relevance and Contribution

We are not aware of a previously published RAG architecture targeted specifically at post-surgical cardiovascular decision support; the architecture described here is intended to fill that gap. The central contribution is not the RAG framework itself, which is well established in natural language processing, but rather its adaptation to the data types, evidence sources, and clinical constraints of cardiac surgery. Evidence comes from heterogeneous sources (procedure-specific outcome registries, surgical guidelines, intraoperative event logs, and continuous physiological telemetry); the relevant time horizon shifts hour by hour; and the cost of a missed deterioration is high enough that auto-resolving evidence conflicts is not an acceptable design choice. The augmentation layer’s combination of a stacked ensemble with confidence interval reporting, temporal reweighting tied to postoperative day, and surfaced (rather than silenced) evidence conflicts does not, on a non-systematic review of related work, appear together in published cardiovascular monitoring systems. The three-layer design makes the reasoning process transparent: clinicians can see which retrieved cases and guidelines shaped each recommendation, rather than receiving an unexplained risk score.

In post-surgical care, the delayed recognition of deterioration has measurable consequences for mortality and morbidity. Interpretability is not only a regulatory requirement in this context—it is a clinical one. Surgeons and intensivists are more likely to act on a recommendation when they can see why it was made and which patients or outcomes it draws on.

### 4.2. Architectural Contributions

The architectural contribution is the combination, within a single workflow, of established techniques chosen for this clinical setting: domain-adapted dense vector retrieval over cardiovascular surgery literature and institutional outcome data; ensemble risk modelling that produces calibrated probability estimates rather than free-text prose; and grounded language generation with citation provenance. The 768-dimensional semantic embedding approach enables matching between clinical scenarios and relevant evidence beyond keyword-based search. The context-aware evidence integration addresses the challenge of personalising population-level evidence to individual patient characteristics, a critical requirement for effective clinical decision support.

Integrating multiple knowledge sources—published literature, guideline repositories, and institutional outcome data—requires active quality management; contradictory evidence between sources is surfaced rather than silently resolved. The provenance tracking built into the generative layer means that every output carries a citation trail, which also supports post hoc audits if a recommendation is later questioned.

### 4.3. Evaluation Approach and Path to Clinical Validation

The proposed evaluation methodology provides a structured approach to assessing RAG-based clinical decision support systems, addressing both technical performance and clinical applicability. While this initial evaluation focuses on system design validation and expert assessment, it establishes a foundation for comprehensive clinical validation studies necessary for widespread deployment.

The emphasis on interpretability assessment and safety evaluation reflects the unique requirements of healthcare AI applications, where system transparency and risk management are paramount. The evaluation framework could serve as a template for assessing other clinical AI applications, providing standardised approaches to technical validation, clinical relevance assessment, and safety evaluation.

### 4.4. Implementation and Future Work

The modular architecture design facilitates gradual implementation and iterative refinement based on clinical feedback and performance assessment. The system’s integration with existing electronic health record systems and clinical workflows aims to support practical feasibility while minimising disruption to established care processes. Future development priorities include expansion to additional clinical specialties, integration with real-time physiological monitoring systems, and the development of adaptive learning mechanisms that continuously improve based on clinical outcomes.

The evaluation methodology provides a pathway for systematic validation and refinement. Future priorities include multi-institutional validation studies, cost-effectiveness analysis, and investigation of long-term impact on clinical outcomes.

### 4.5. Limitations and Considerations

Several limitations should be acknowledged in this system design and evaluation approach. The focus on post-surgical cardiovascular monitoring may limit the immediate generalisability to other clinical domains, though the architectural principles could be adapted for broader applications. The evaluation methodology is detailed but resource-intensive; it will require close collaboration with clinical sites and cannot be fully executed within a single-centre study.

The absence of actual clinical deployment results limits conclusions about real-world effectiveness and safety, highlighting the need for prospective validation studies. The system’s dependence on high-quality clinical data and expert annotation for optimal performance represents an implementation consideration that must be addressed through robust data governance and quality assurance procedures.

## 5. Conclusions

We have described a RAG-based clinical decision support system for post-surgical cardiovascular monitoring, with a design emphasis on evidence traceability and workflow compatibility. The three-layer architecture—retrieval, augmentation, and generative—allows each component to be validated and refined independently, and the proposed evaluation framework provides concrete performance targets across technical, clinical, and safety dimensions.

The system is not yet clinically deployed, and the evaluation framework described here sets out what will need to be demonstrated before that step is taken. The next phase of this work will involve single-site piloting alongside existing systems, with the primary aim of establishing whether the AI-generated recommendations are accurate enough and fast enough to be useful in a real post-surgical setting. If that evidence is positive, expansion to additional centres and cardiac surgery subspecialties will follow.

The proposed evaluation framework sets concrete aspirational benchmarks: retrieval precision >90% for relevant evidence—a deliberately high threshold reflecting the safety requirement that evidence cited in deterioration recommendations should rarely be misleading. This exceeds what current open clinical-NLP information-retrieval benchmarks achieve, where state-of-the-art precision in the top 10 systems sits at approximately 0.30–0.40 [26] (best automatic system in the 2016 CDS Track: P@10 = 0.40), and would need to be approached through tight domain restriction, similarity threshold filtering, and post-retrieval reranking rather than open IR. A query response time <3 s was chosen because longer latencies are known to disrupt bedside workflow in intensive-care settings, and a clinical appropriateness rating >85% from expert review of generated recommendations is a threshold consistent with prospective evaluations of decision support tools that have proceeded to pilot deployment. These figures are aspirational targets, not measured outcomes; meeting them is a precondition for pilot deployment rather than a claim about current performance.

## Figures and Tables

**Table 1:** Data sources for the post-surgical monitoring RAG system.

Preoperative Phase	Intraoperative Phase	Postoperative Phase
Structured data from electronic health records (EHRs): cardiac history, comorbidities (diabetes, hypertension, renal dysfunction), medications, baseline cardiac function (ejection fraction, valve function)	Surgical procedure data: type of surgery (CABG, valve replacement, combination), CPB time, aortic cross-clamp time	Vital Signs: Wearable continuous telemetry (ECG, SpO_2_, RR, temperature, blood pressure). Integration with CAREPOI (™) perioperative telemetry monitoring system via HL7/FHIR. Early detection of haemodynamic instability, arrhythmias, and respiratory compromise.
Preoperative diagnostic tests: ECG, echocardiography, cardiac catheterization, stress tests, laboratory values	Intraoperative events: arrhythmias, haemodynamic instability, transfusion requirements/haemodynamic parameters during surgery/laboratory trend monitoring: troponin, lactate, creatinine/serial biochemical markers: troponin, BNP/NT-proBNP, lactate, creatinine, inflammatory markers	Alerts and Early Warning Scores: Clinical deterioration early warning scores (NEWS2, MEWS) computed continuously from wearable telemetry. DETER-type algorithms [16] provide real-time deterioration risk stratification, triggering escalation pathways and clinician notifications.
Risk assessment scores: EuroSCORE II, STS Predicted Risk of Mortality and Morbidity	Continuous biosignal monitoring from ICU/step-down telemetry: ECG (continuous), arterial blood pressure (invasive/non-invasive), cardiac output monitoring, central venous pressure, pulmonary artery pressure (when available)	AI-Driven Deterioration Prediction: DETER-type algorithms [16] combining device-based biosignal telemetry with generative AI for real-time composite risk scoring; post-discharge readmission prediction; integration of troponin, BNP/NT-proBNP, and creatinine trends into deterioration models.
Patient-reported symptoms and functional status/medication administration records	Clinical assessments: physician and nursing notes, chest tube output, urine output, fluid balance/imaging: chest X-rays, echocardiography when indicated/anaesthesia records and intraoperative monitoring data	Complication Detection and NLP Processing: SSI, DVT, sepsis, AKI detected via NLP on free text (BioBERT [19], cTAKES [20]). Patient-reported symptoms (chest pain, dyspnoea, palpitations) extracted from recovery questionnaires and telehealth interactions. Wearable activity monitors and patient-reported outcome measures (PROMs) integrated for functional recovery tracking.

CABG: coronary artery bypass grafting; CPB: cardiopulmonary bypass; ECG: electrocardiogram; SpO_2_: peripheral oxygen saturation; RR: respiratory rate; HL7: Health Level Seven; FHIR: Fast Healthcare Interoperability Resources; ICD: International Classification of Diseases; NEWS2: National Early Warning Score 2; MEWS: Modified Early Warning Score; SSI: surgical site infection; DVT: deep vein thrombosis; AKI: acute kidney injury; NLP: natural language processing; BNP: B-type natriuretic peptide; NT-proBNP: N-terminal proBNP; PROM: patient-reported outcome measure.

**Table 2:** Proposed configuration of the three-layer RAG architecture. Values represent planned settings for pilot deployment; empirical hyperparameter tuning will follow clinical validation.

Layer/Component	Configuration	Notes
Retrieval—embedding model	Sentence-BERT (768-dim); ClinicalBERT optional for clinical-note retrieval	Encoder-only; used for similarity search, not generation
Retrieval—similarity metric	Cosine similarity	Standard for dense vector retrieval
Retrieval—top-k	Configurable per query type	Tuned during pilot evaluation
Augmentation—ensemble	Stacking ensemble: Random Forest + XGBoost base learners; logistic-regression meta-learner	Outputs calibrated probabilities
Augmentation—calibration	Probability calibration via meta-learner; confidence intervals reported	Specific calibration method to be selected during evaluation
Augmentation—outcome labels	30-day mortality, MACE, readmission, functional recovery (planned)	Prototype uses MIMIC-III mortality + ICD-coded events
Generative—base model	Domain-fine-tuned BERT classifier (discriminative)	Procedure-specific complication probabilities
Generative—downstream LLM	Gemini 2.5 Pro	Receives top-k retrieved evidence + augmentation outputs as prompt
Generative—alignment	Supervised fine-tuning + RLHF (planned)	Once expert-curated feedback dataset is available
Generative—hallucination control	Retrieval-restricted prompt; suppression below similarity threshold; post hoc claim verification	Falls back to augmentation-layer output if generation fails verification
Generative—evidence reconciliation	Multi-source conflicts surfaced rather than silently selected	Clinical judgement resolves the disagreement

## Data Availability

As this is a system design study, no new clinical datasets were generated. Prototype evaluation used publicly available, de-identified data from the MIMIC-III Clinical Database [21] and the MIMIC-III-Ext-PPG benchmark [22], both available via PhysioNet (https://physionet.org/) under their respective data use agreements. Future validation studies will make anonymised data from participating clinical sites available through appropriate repositories in accordance with institutional, GDPR, and (where applicable) HIPAA requirements.
